# Osteoarthritis Is Associated With an Increased Risk of Age-Related Macular Degeneration: A Population-Based Longitudinal Follow-Up Study

**DOI:** 10.3389/fmed.2022.854629

**Published:** 2022-05-10

**Authors:** Yi-Hsiang Chiu, Jehn-Yu Huang, Ya-Ping Huang, Shin-Liang Pan

**Affiliations:** ^1^Department of Physical Medicine and Rehabilitation, National Taiwan University Hospital, Taipei, Taiwan; ^2^Department of Ophthalmology, National Taiwan University Hospital, Taipei, Taiwan; ^3^Department of Physical Medicine and Rehabilitation, College of Medicine, National Taiwan University, Taipei, Taiwan; ^4^Department of Physical Medicine and Rehabilitation, National Taiwan University Hospital, Yun-Lin Branch, Yun-Lin County, Taiwan

**Keywords:** epidemiology study, inflammation, macular degeneration, osteoarthritis, risk factor

## Abstract

**Aims:**

To investigate the long-term risk of age-related macular degeneration (AMD) in persons with osteoarthritis (OA).

**Methods:**

This retrospective cohort study first enrolled 71,609 subjects diagnosed with OA, and 236,169 without such a diagnosis between January 1, 2002 and December 31, 2005, from the Longitudinal Health Insurance Database 2005. All were aged 40–69. After excluding subjects who had pre-existing AMD and/or who had missing socioeconomic data, frequency matching by sex and age was performed. This resulted in there being 60,274 subjects in each of the final matched OA and non-OA groups. The study participants were followed up to the occurrence of AMD, death, or the end of 2011. We used Cox proportional-hazards regression to estimate the impact of OA on the risk of developing AMD, and performed subgroup analyses stratified by sex and age.

**Results:**

The median follow-up time was 8.9 years, with an interquartile range of 1.4 years. The incidence rate of AMD in the OA group was 2.77 per 1,000 person-years [95% confidence interval (CI), 2.62–2.92], and in the non-OA group, 2.06 per 1,000 person-years (95% CI, 1.94–2.19). The adjusted hazard ratio (HR) of AMD for the OA group was therefore 1.30 (95% CI, 1.20–1.41). In the subgroup analysis stratified by sex for the OA group, the adjusted HRs of AMD were 1.29 in the women's stratum and 1.31 in the men's. When stratified by age, the adjusted HRs of AMD for the younger (40–54 years) and older (55–69 years) strata were 1.28 and 1.31, respectively.

**Conclusions:**

Persons with OA have an increased risk of developing AMD, regardless of age and sex.

## Introduction

Age-related macular degeneration (AMD), the leading cause of vision loss in people over age 50, is a progressive degenerative disease of the central retina (macula) (1). AMD can cause functional disabilities and a higher risk of fall-related injuries, leading to enormous socioeconomic and healthcare burdens (2). Therefore, it is imperative to identify the risk factors of AMD. Aside from aging, the risk factors for AMD include smoking, cardiovascular diseases, and genetic predisposition (3, 4). In addition, it has been suggested that chronic inflammation participates in AMD's pathogenesis (5). Osteoarthritis (OA), the most common form of arthritis, is characterized by cartilage degeneration and synovial inflammation (6). Because inflammation may play a role in both OA and AMD, researchers have examined the association between these two conditions. Two case-control studies reported an increased risk of arthritis in people with early or neovascular AMD (7, 8). In addition, one cohort study indicated a slightly increased risk of AMD after OA [hazard ratio (HR) = 1.06] (9). However, one cross-sectional study suggested that arthritis was not associated with AMD (10). And one recent cross-sectional study even reported that people with AMD were less likely to have OA than their non-AMD-patient counterparts (odds ratio = 0.43) (11). In short, the association between OA and AMD remains controversial. Moreover, because these previous studies mainly had case-control or cross-sectional designs without longitudinal follow-up, the temporal relationship between OA and AMD is largely unexplored. Therefore, to fill this gap, we conducted the present population-based, longitudinal follow-up study to investigate the long-term risk of AMD in patients with OA.

## Materials and Methods

### Data Sources

Implemented in 1995, Taiwan's National Health Insurance (NHI) is compulsory for all 22 million citizens, and covers 99% of the population. This study used the Longitudinal Health Insurance Database 2005 (LHID2005), which consists of 1 million insurance claimants randomly selected from the complete NHI research database. LHID2005 has been verified as a representative of the original NHI database (https://nhird.nhri.org.tw/date_01_en.html). The personally identifiable information in this database has been encrypted to protect the privacy of research participants. This study was approved by the Research Ethics Committee of National Taiwan University Hospital (NTUH-REC No.: 202109143W).

### Study Design

**Figure 1:**
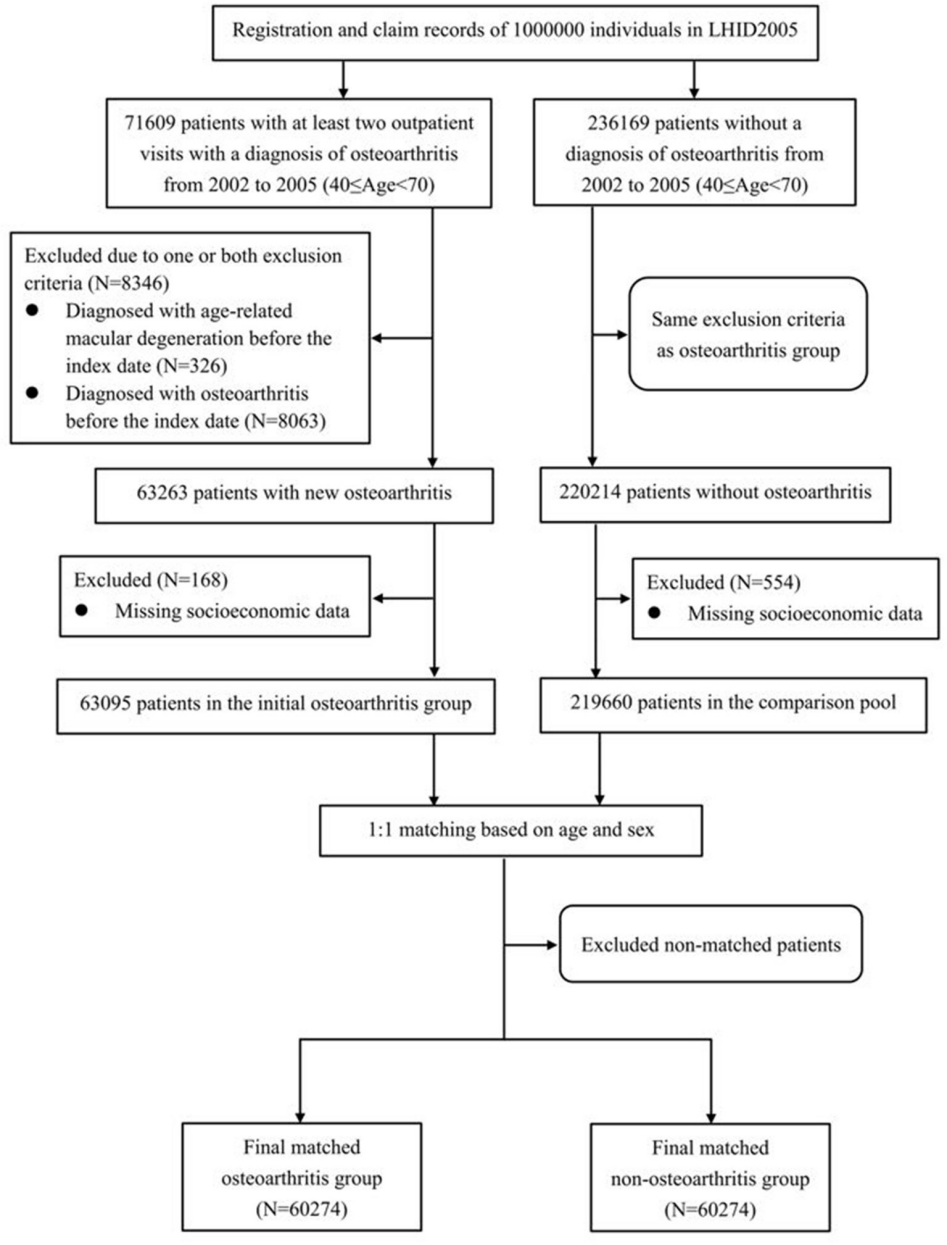
Flowchart illustrating the enrollment process of the osteoarthritis and non-osteoarthritis groups.

This study adopted a retrospective cohort study design. The study population consisted of an OA group and a non-OA group. The enrollment processes of these two groups are shown in Figure 1. The initial inclusion criteria for the OA group were: (1) at least two outpatient visits with a diagnosis of OA [International Classification of Diseases, 9th edition, clinical revision (ICD-9-CM) code 715] between January 1, 2002 and December 31, 2005, with the date of the first OA diagnosis of each subject defined as that person's index date; and (2) being 40–69 years old on the index date. These inclusion criteria resulted in 71,609 persons being selected for the initial OA group. Then, we excluded members of that group who (1) had been diagnosed with AMD (ICD-9-CM codes 362.50, 362.51, or 362.52) before their respective index dates; and/or (2) had been diagnosed with OA (ICD-9-CM code 715) before their index dates to maximize the likelihood of including newly diagnosed OA cases. This led to a total of 8,346 participants being removed from the initial OA group (Figure 1).

Baseline comorbidities and socioeconomic factors were included in our analysis of the association between OA and AMD. The selected comorbid conditions were diabetes (ICD-9-CM code 250), hypertension (ICD-9-CM codes 401–405), dyslipidemia (ICD-9-CM code 272), coronary heart disease (ICD-9-CM codes 410–414 and 429.2), stroke (ICD-9-CM codes 430–438), chronic obstructive pulmonary disease (ICD-9-CM codes 491, 492, and 496) and chronic kidney disease (ICD-9-CM codes 580–587). A given comorbidity was deemed to be present if at least two outpatient visits or one inpatient discharge record contained the relevant diagnostic code(s).

The socioeconomic factors included in the analysis were (1) the geographic location of the participant's residence (northern, central, eastern, or southern Taiwan); (2) the level of urbanization at his/her main residence; and (3) his/her monthly income. The townships were categorized into seven levels of urbanization, of which level 1 represents the highest level of urbanization, and level 7 represents the lowest level of urbanization (12). In the present study, the level 5, 6, or 7 were merged into a single level 5 owing to few participants in these three levels. The payroll data recorded in the NHI for insurance purposes were used to as indicators of the subjects' monthly incomes, which were divided into four levels: (1) New Taiwan Dollars (NT$) 0, (2) NT$1–NT$15,840, (3) NT$15,841–NT$25,000, and (4) ≥NT$25,001. NT$15,840 was selected as the first cut-off because it was the monthly income of full-time minimum-wage employees in Taiwan throughout the data-collection window. The OA patients with missing data on any of the above-mentioned socioeconomic factors (*n* = 168) were excluded from the study. Thus, the final OA group comprised 63,095 subjects (Figure 1).

The inclusion criteria for the non-OA group were (1) not having been diagnosed with OA during outpatient visits between January 1, 2002 and December 31, 2005, with the subject's first outpatient visit during this period being defined as the index date; and (2) being 40–69 years old at the index date. We initially included 236,169 non-OA subjects. The exclusion criteria for the non-OA group were the same as those of the OA group. In addition, the persons with missing data on socioeconomic factors were excluded (*n* = 554) (Figure 1). After those exclusion criteria were applied, 219,660 participants remained in the non-OA control pool. We then frequency-matched each OA subject with one randomly selected non-OA subject of the same sex and age. After frequency matching, there were 60,274 subjects in each of the final OA and non-OA groups.

### Outcome

The outcome was the first diagnosis of AMD during follow-up. Each subject was tracked from his/her index date until (1) the earliest occurrence of AMD, as determined based on at least one discharge record or at least two outpatient-visit records with an AMD diagnosis (ICD-9-CM codes 362.50, 362.51, or 362.52); (2) death; or (3) the end of 2011, whichever occurred first. For each subject who died, the cause of death was deemed to be the same as the main diagnostic code associated with discharge or outpatient visits in the final 3 months of his/her life (13).

### Statistical Analysis

Chi-square tests and Student's *t*-tests were applied to the differences in baseline characteristics between the OA group and the non-OA group. The incidence rate was calculated as the number of AMD cases divided by the total amount of AMD-free follow-up time per 1,000 person-years. The cumulative incidence of AMD for the OA and non-OA groups was estimated using the Kaplan-Meier method, and compared using log-rank testing. We used Cox proportional-hazards regression to estimate the impact of OA on the risk of developing AMD, and performed subgroup analyses stratified by sex and age. The proportional hazards assumption was tested using the Schoenfeld residuals, with no violation detected. To evaluate the impact of sex differences on the association between OA and AMD, we performed analyses that included an interaction term (i.e., the product of sex and OA in the regression model) for sex-stratified analysis. For testing the influence of age in the age-stratified analysis, we included an interaction term as the product of age and OA in the regression analysis. All statistical analyses were performed using SAS version 9.4 software (SAS Institute, Cary, NC), and the statistical significance was set to alpha level = 0.05.

## Results

Table 1 presents the distributions of baseline socioeconomic and clinical characteristics for the OA and non-OA groups. The mean ages of the OA and non-OA groups were both 54.8 years. Of the two, the OA group exhibited higher rates of all seven of the comorbidities, and slightly more than double the rate, in the case of dyslipidemia. There were also significant differences in the two groups' distributions of monthly income, urbanization level, and geographic location.

**Table 1:** Baseline demographic and socioeconomic characteristics and comorbid conditions of the osteoarthritis (OA) and non-OA groups.

**Variable**	**OA group (*n* = 60,274)**	**Non-OA group (*n* = 60,274)**	***p-*value**
Female	36,760 (61.0%)	36,760 (61.0%)	1.0000
Age, years	54.8 (8.1)	54.8 (8.2)	0.7125
Diabetes	6,379 (10.6%)	4,457 (7.4%)	<0.0001
Hypertension	14,261 (23.7%)	9,336 (15.5%)	<0.0001
Dyslipidemia	6,323 (10.5%)	3,160 (5.2%)	<0.0001
Coronary heart disease	4,289 (7.1%)	2,626 (4.4%)	<0.0001
Stroke	2,284 (3.8%)	1,533 (2.5%)	<0.0001
COPD	604 (1.0%)	443 (0.7%)	0.0001
Chronic kidney disease	1,227 (2.0%)	823 (1.4%)	<0.0001
Monthly income			<0.0001
NT$0	17,080 (28.3%)	15,005 (24.9%)	
NT$1–NT$15840	9,099 (15.1%)	8,481 (14.1%)	
NT$15841–NT$25000	20,533 (34.1%)	21,680 (36.0%)	
≧NT$25001	13,562 (22.5%)	15,108 (25.0%)	
Urbanization level			<0.0001
1 (most urbanized)	25,523 (42.3%)	26,857 (44.6%)	
2	18,379 (30.5%)	17,315 (28.7%)	
3	5,213 (8.7%)	5,815 (9.7%)	
4	6,024 (10.0%)	5,866 (9.7%)	
5 (least urbanized)	5,135 (8.5%)	4,421 (7.3%)	
Geographic region			<0.0001
Northern	30,637 (50.8%)	31,361 (52.0%)	
Central	12,723 (21.1%)	12,382 (20.5%)	
Southern	15,413 (25.6%)	15,458 (25.7%)	
Eastern	1,501 (2.5%)	1,073 (1.8%)	

*Values are expressed as mean ± SD or n (%)*.

*NT$, New Taiwan Dollar; COPD, chronic obstructive pulmonary disease*.

*US$1 = NT$34 in 2001*.

**Figure 2:**
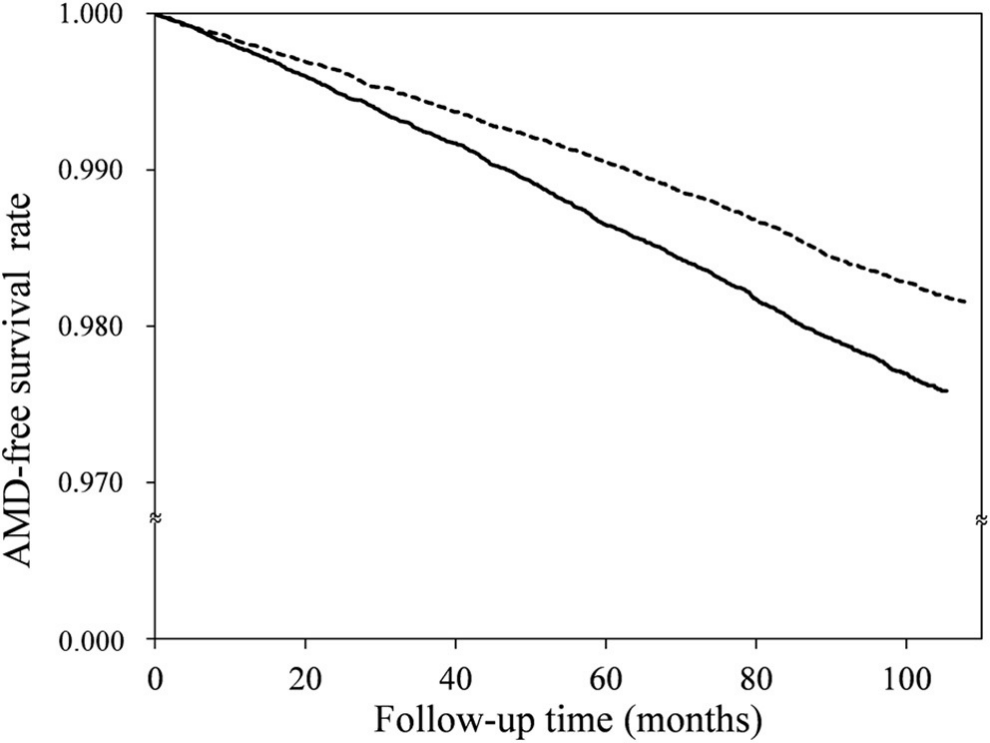
Age-related macular degeneration (AMD)-free survival rates for the osteoarthritis (solid line) and non-osteoarthritis (dotted line) groups.

The median follow-up time was 8.9 years, with an interquartile range of 1.4 years. The number of AMD cases, and the HRs for the OA and non-OA groups, are presented in Table 2. Among the 60,274 OA patients, 1,280 cases of AMD occurred over 462,797.5 person-years, an incidence rate of 2.77/1,000 person-years [95% confidence interval (CI), 2.62–2.92]. Among the 60,274 patients without OA, on the other hand, there were 1,069 cases of AMD over 518,354.5 person-years: an incidence rate of 2.06/1,000 person-years (95% CI, 1.94–2.19). Thus, as compared with the non-OA group, the adjusted HR of AMD for the OA group was 1.30 (95% CI, 1.20–1.41, *p* < 0.0001). In addition, the AMD-free survival rate of the OA group was lower than that of the non-OA group (Figure 2, *p* < 0.0001).

**Table 2:** Number of age-related macular degeneration (AMD) events and hazard ratios (HRs) for the osteoarthritis (OA) and non-OA groups.

**Variable**	**Non-OA group (*n* = 60,274)**	**OA group (*n* = 60,274)**
AMD events, *n*	1,069	1,280
Risk per 1,000 person-years (95% CI)	2.06 (1.94–2.19)	2.77 (2.62–2.92)
Crude HR (95% CI)	1.00	1.34 (1.24–1.46)^a^
Adjusted HR^b^ (95% CI)	1.00	1.30 (1.20–1.41)^a^

a *p < 0.05*.

b *Adjusted for sex, age, diabetes, hypertension, dyslipidemia, coronary heart disease, stroke, chronic obstructive pulmonary disease, chronic kidney disease, monthly income, urbanization level, and geographic region*.

*AMD, age-related macular degeneration; CI, confidential interval; HR, hazard ratio; OA, osteoarthritis*.

Table 3 present the results of subgroup analysis of AMD risk, stratified by sex and age, respectively. In the former, the adjusted HRs of AMD for the OA group in the women's and men's strata were 1.29 and 1.31, respectively. In other words, women and men with OA had similarly greater magnitudes of AMD risk than their respective non-OA counterparts. Meanwhile, it can be seen that the younger (40–54 years) and older (55–69 years) strata had similar point estimates of adjusted AMD HR (1.28 and 1.31, respectively), although the 95% CI was wider for the younger stratum. The interaction tests showed no significant interaction effect of sex (*p* = 0.8617) and no significant interaction effect of age (*p* = 0.5068) on the association between OA and AMD.

**Table 3:** Crude and adjusted hazard ratios (HRs) of age-related macular degeneration (AMD) for patients with osteoarthritis (OA), stratified by sex and age.

	**Women**		**Men**		**40** **≤age** **<** **55**		**55** **≤age** **<** **70**	
**Variable**	**Non-OA group (*n* = 36,760)**	**OA group (*n* = 36,760)**	**Non-OA group (*n* = 23,514)**	**OA group (*n* = 23,514)**	**Non-OA group (*n* = 32,162)**	**OA group (*n* = 32,162)**	**Non-OA group (*n* = 28,112)**	**OA group (*n* = 28,112)**
AMD events, n	668	796	401	484	241	279	828	1,001
Risk per 1,000 person-years (95% CI)	2.10 (1.94–2.26)	2.79 (2.60–2.99)	2.01 (1.82–2.22)	2.73 (2.50–2.99)	0.86 (0.75–0.97)	1.11 (0.99–1.25)	3.48 (3.25–3.73)	4.72 (4.43–5.02)
Crude HR (95% CI)	1.00	1.33 (1.20–1.48)^a^	1.00	1.36 (1.19–1.56)^a^	1.00	1.33 (1.12–1.59)^a^	1.00	1.35 (1.23–1.48)^a^
Adjusted HR^b^ (95% CI)	1.00	1.29 (1.16–1.44)^a^	1.00	1.31 (1.15–1.50)^a^	1.00	1.28 (1.07–1.52)^a^	1.00	1.31 (1.19–1.44)^a^

a *p < 0.05*.

b *Adjusted for sex, age, diabetes, hypertension, dyslipidemia, coronary heart disease, stroke, chronic obstructive pulmonary disease, chronic kidney disease, monthly income, urbanization level, and geographic region*.

*AMD, age-related macular degeneration; CI, confidential interval; HR, hazard ratio; OA, osteoarthritis*.

We carried out a sensitivity analysis to adjust for other rheumatic diseases, including rheumatoid arthritis (ICD-9-CM code 714.0), gout (ICD-9-CM code 274), seronegative arthritis (ICD-9-CM code 720), systemic lupus erythematosus (ICD-9-CM code 710.0), in addition to the covariates listed in Table 1 in the Cox regression analysis. As a result, the estimated adjusted HR was 1.34 (95% CI, 1.23–1.46), which is close to the original analyses' results (adjusted HR 1.30, 95% CI, 1.20–1.41).

## Discussion

This study of the association between OA and long-term risk of AMD, based on a nationally representative Taiwanese sample, yielded the following three main findings. First, patients with OA had a 30% higher risk of AMD than those without OA. Second, elevation in the risk of AMD among OA patients was similar for both men and women (adjusted HRs = 1.29 and 1.31, respectively). Third, the propensity to suffer from AMD was similar across the younger subjects with OA (i.e., 40–54 years old, adjusted HR = 1.28) and older ones (55–69 years old, adjusted HR = 1.31). Although the mechanisms underlying the positive temporal association between OA and AMD remain unclear, we propose the following possible explanations.

AMD is classified into non-exudative (dry) and exudative (wet) types (2). The pathological findings of dry AMD include geographic atrophy of the retinal pigment epithelium (RPE) and subretinal drusen deposits (2, 14). The pathogenetic mechanisms of dry AMD are not well understood. Previous studies have suggested decreased perfusion of the choroid and Bruch's membrane in dry AMD, leading to ischemia of photoreceptor and RPE (15). Besides, dry AMD is also related to RPE cell apoptosis which may be induced by oxidative and inflammatory stress (15). On the other hand, wet AMD is characterized by choroidal neovascularization (2, 16). The development of wet AMD is related to an increased level of vascular endothelial growth factor (VEGF) in the vitreous and retina (2, 17). VEGF elicits endothelial cells migration and de novo blood vessel formation. The newly formed immature vessels grow toward the outer retina from the underlying choroid, and then fluid leaking within or below the retina from these immature vessels (17). Recent research has shown that chronic inflammation and inflammatory mediators play important roles in the initiation and progression of OA (18, 19). Increased circulating levels of interleukin-6 (IL-6) and tumor necrosis factor-α, both of which are key pro-inflammatory cytokines, have been associated with cartilage loss in patients with OA (20, 21). Moreover, elevated serum C-reactive protein (CRP), a systemic inflammatory marker, has been found in patients with OA (22), and such elevated CRP levels may predict the progression of OA (23). Inflammation has also been postulated to have a role in the pathogenesis of AMD (24, 25). Prior studies have shown that patients with AMD have elevated levels of circulating inflammatory markers, including but not limited to CRP and IL-6 (26–28), suggesting that chronic inflammation may be related to an increased risk of AMD (26). Inflammatory cytokines can enhance the secretion of vascular endothelial growth factor, which can cause retinal and choroidal neovascularization in AMD (29). We therefore hypothesize that the observed higher AMD risk among patients with OA may be mediated by chronic inflammation, which predisposes them to this higher risk.

Also, individuals who develop OA can become physically inactive due to joint pain (30). Previous research has shown that physical inactivity is associated with a higher risk of both early and late AMD (31), as well as with increased systemic oxidative stress and endothelial dysfunction (32). And oxidative stress and choroidal vascular dysfunction may be involved in the pathogenesis of AMD (33, 34). Therefore, we propose that OA-related physical inactivity may increase oxidative stress, which in turn leads to a higher risk of developing AMD.

Epidemiological studies have revealed a high prevalence of vitamin D deficiency in patients with OA (35, 36). Although the role of vitamin D deficiency in OA remains uncertain, lower levels of vitamin D have been linked to decreases in cartilage thickness (37). On the other hand, observational studies have shown that vitamin D deficiency may be a potential risk factor for AMD (38, 39). In addition, vitamin D may reduce the risk of developing AMD by protecting against oxidative stress, inhibiting inflammation and amyloid deposits, and combating angiogenesis (39). Accordingly, the increased risk of AMD in OA patients may also be partly explained by the OA-related vitamin D deficiency.

In this study, we found that the magnitudes of the HRs for AMD between women and men with OA were similar, as were those of younger and older OA patients. People with OA may have impaired balance and difficulty in walking, due to joint pain being exacerbated by movement. Thus, when OA and the impaired visual acuity brought about by AMD are present in the same individual, s/he could be especially prone to falling (40). As such, clinicians caring for OA patients should be alert to their increased risk of AMD regardless of sex and age, and periodically access their balance function as well as their visual acuity.

The chief strength of the present study is its use of a nationally representative, large-scale longitudinal database, which enabled us to identify all new AMD cases in OA patients during the follow-up period. The NHI program is a single-payer compulsory social insurance plan with a very high coverage rate in Taiwan. The barrier to medical access is negligible because the NHI system allows patients to visit any clinic or hospital freely without referral by a general practitioner, and patients pay only about $5–$15 USD at each visit. Considering the minimal barrier to medical access in Taiwan, it can be expected that most patients who developed AMD would seek medical help and would be captured in the NHI database, which enabled us to identify all incident cases of AMD and establish a temporal relationship between OA and AMD.

However, this study has several limitations that should be acknowledged. First, the diagnoses of OA, AMD, and medical comorbidities were determined entirely using the ICD codes from the LHID2005, a subset of the NHI database. The information of the image findings is not available in the LHID2005 database. This may raise concerns about diagnostic accuracy. However, the NHI Bureau's audit committees randomly sample claims data and review medical records to verify such accuracy, as well as the quality of care provided. There are differences in the sensitivity and positive predictive value of diagnosis across multiple conditions from the NHI database (41). Lin et al. used ophthalmoscopic findings and chart review to validate the ICD-9-CM codes of AMD in the NHI database, and reported that the positive predictive value is 0.96 (95% confidence interval, 0.95–1.01) (42), which may indicate acceptable diagnostic accuracy of AMD in the LHID2005. Nevertheless, as the majority of the AMD diagnoses were coded as unspecified AMD (ICD-9-CM code 362.50), the subtype analysis (i.e., non-exudative and exudative AMD) was not carried out in this study.

Second, we did not evaluate the influence of OA severity and medications (e.g., non-steroidal anti-inflammatory drugs, NSAIDs) on the risk of AMD. Since NSAIDs have anti-inflammatory effects, the use of NSAIDs may influence the association between OA and AMD if chronic inflammation is one of the underlying mechanisms. Nevertheless, NSAIDs are commonly used in most OA patients, without the possibility of being separated from the biological plausibility resulting from OA by using the NHI database. Moreover, NSAIDs are widely available as over-the-counter drugs, so the use of NSAIDs may not be readily measurable using the insurance database. Future studies are needed to examine the relationship between OA severity and the risk of AMD, and evaluate whether using NSAIDs to inhibit the inflammatory pathways can reduce the risk of AMD.

Third, information on specific risk factors of AMD, such as genetic factors and lifestyle (e.g., smoking), is not available in the LHID2005 and not included in the analysis. Therefore, there may be residual confounding on the association between OA and AMD. Fourth, the study included only the Taiwanese population. Whether the results can apply to other ethnic groups deserves further investigation.

In summary, the present population-based longitudinal follow-up study demonstrated that Taiwanese patients with OA are at a higher risk of developing AMD, regardless of age and sex. Our findings suggest that clinicians who care for OA patients should be vigilant regarding this risk.

## Data Availability Statement

The original contributions presented in the study are included in the article/supplementary materials, further inquiries can be directed to the corresponding author.

## Ethics Statement

The studies involving human participants were reviewed and approved by Research Ethics Committee of National Taiwan University Hospital. Written informed consent for participation was not required for this study in accordance with the national legislation and the institutional requirements.

## Author Contributions

J-YH, Y-PH, and S-LP designed the research. Y-PH, Y-HC, and S-LP conducted the research. Y-PH and S-LP analyzed data. Y-HC, J-YH, Y-PH, and S-LP wrote the manuscript and had primary responsibility for final content. All authors read and approved the final manuscript.

## Conflict of Interest

The authors declare that the research was conducted in the absence of any commercial or financial relationships that could be construed as a potential conflict of interest.

## Publisher's Note

All claims expressed in this article are solely those of the authors and do not necessarily represent those of their affiliated organizations, or those of the publisher, the editors and the reviewers. Any product that may be evaluated in this article, or claim that may be made by its manufacturer, is not guaranteed or endorsed by the publisher.
